# Atrophy patterns in isolated subscapularis lesions

**DOI:** 10.1186/s12891-021-04241-5

**Published:** 2021-04-22

**Authors:** Gernot Seppel, Andreas Voss, Daniel J. H. Henderson, Simone Waldt, Bernhard Haller, Philipp Forkel, Sven Reuter, Boris M. Holzapfel, Johannes E. Plath, Andreas B. Imhoff

**Affiliations:** 1 Department of Orthopedic Sports Medicine, Klinikum rechts der Isar, Technical University of Munich, Ismaninger Str. 22, 81675 Munich, Germany; 2 Clinic for Orthopedics and Sports Orthopedics, OSP München, Munich, Germany; 3 Department of Trauma Surgery, University Hospital Regensburg, Regensburg, Germany; 4 Department of Orthopedics, Leeds General Infirmary, Great George Street, Leeds, UK; 5 Department of Radiology, Klinikum rechts der Isar, Technical University of Munich, Munich, Germany; 6 Institute of Medical Informatics, Statistics and Epidemiology, Klinikum rechts der Isar, Technical University of Munich, Munich, Germany; 7 SRH University of Applied Health Sciences, Stuttgart, Germany; 8 Department of Orthopedics, König Ludwig Haus, University of Würzburg, Würzburg, Germany; 9 Department of Trauma, Orthopedics, Plastic and Hand Surgery, Zentralklinikum Augsburg, Augsburg, Germany

**Keywords:** Isolated subscapularis atrophy, Subscapularis repair, Rotator cuff, Rotator cuff atrophy, Subscapularis atrophy

## Abstract

**Background:**

While supraspinatus atrophy can be described according to the system of Zanetti or Thomazeau there is still a lack of characterization of isolated subscapularis muscle atrophy. The aim of this study was to describe patterns of muscle atrophy following repair of isolated subscapularis (SSC) tendon.

**Methods:**

Forty-nine control shoulder MRI scans, without rotator cuff pathology, atrophy or fatty infiltration, were prospectively evaluated and subscapularis diameters as well as cross sectional areas (complete and upper half) were assessed in a standardized oblique sagittal plane. Calculation of the ratio between the upper half of the cross sectional area (CSA) and the total CSA was performed. Eleven MRI scans of patients with subscapularis atrophy following isolated subscapularis tendon tears were analysed and cross sectional area ratio (upper half /total) determined. To guarantee reliable measurement of the CSA and its ratio, bony landmarks were also defined.

All parameters were statistically compared for inter-rater reliability, reproducibility and capacity to quantify subscapularis atrophy.

**Results:**

The mean age in the control group was 49.7 years (± 15.0).

The mean cross sectional area (CSA) was 2367.0 mm^2^ (± 741.4) for the complete subscapularis muscle and 1048.2 mm^2^ (± 313.3) for the upper half, giving a mean ratio of 0.446 (± 0.046).

In the subscapularis repair group the mean age was 56.7 years (± 9.3). With a mean cross sectional area of 1554.7 mm^2^ (± 419.9) for the complete and of 422.9 mm^2^ (± 173.6) for the upper half of the subscapularis muscle, giving a mean CSA ratio of 0.269 (± 0.065) which was seen to be significantly lower than that of the control group (*p* < 0.05).

**Conclusion:**

Analysis of typical atrophy patterns of the subscapularis muscle demonstrates that the CSA ratio represents a reliable and reproducible assessment tool in quantifying subscapularis atrophy. We propose the classification of subscapularis atrophy as Stage I (mild atrophy) in case of reduction of the cross sectional area ratio < 0.4, Stage II (moderate atrophy) in case of < 0.35 and Stage III (severe atrophy) if < 0.3.

## Background

Prognostic factors in the outcomes of rotator cuff repair have been extensively described and discussed in the literature. Beyond clinical factors such as age, surgeon experience and concomitant diseases, structural factors including tears size and muscle quality seem to have the greatest influence on postoperative outcome [4, 5, 19, 21, 26, 32, 34].

Higher grades of preoperative muscular atrophy and fatty infiltration, specifically, have been demonstrated to result in poorer function and increased re-tear rates post-operatively, and have been shown individually to be independent predictors of outcome [10, 11, 19, 24, 34]. Although muscular atrophy and fatty infiltration may be independent predictors, these properties are clearly linked. Atrophy, describing a decrease in muscle mass, is known to be influenced by fatty infiltration, representing involution of fat between muscle fibers [24].

Although rotator cuff repair is generally recommended to reduce pain and improve shoulder function, proper pre-operative evaluation of fatty infiltration as reported by Goutallier et al. [12] and muscular atrophy on cross sectional imaging is crucial in determining the feasibility of rotator cuff repair during preoperative planning. In addition to muscular retraction as described by Patte et al. [27], muscular atrophy represents an important factor in assessing the feasibility of reconstruction.

In 1996, Thomazeau et al. [35] introduced an MRI classification to quantify supraspinatus atrophy, by calculating the occupation ratio of the muscle within the supraspinatus fossa. Similarly, Zanetti et al. [38] described atrophy of the supraspinatus muscle belly by relation to a tangential line connecting the superior aspect of the coracoid and the scapular spine.

Combined anterosuperior rotator cuff atrophy was characterized by Warner et al. [37], but while Schröder et al. [31] and Scheibel et al. [30] described atrophy of the subscapularis by measuring the vertical, the cranial-transverse and the caudal-transverse diameters as well as the signal to noise ratio of the SSC and the ISP muscle, none of these authors proposed a system of classification.

Isolated subscapularis tendon tears are a rare entity with a prevalence of only 4% among all rotator cuff lesions [7]. Most of the tears appear due to traumatic events like external rotation of the abducted arm in younger patients or hyperextension [8]. while non-traumatic lesions are described as a consequence of subcoracoid impingement [23, 29] or sub−/luxation of the long head of the biceps tendon [36].

The purpose of this study was to further describe atrophy patterns of the subscapularis muscle and to propose a reliable method for quantifying and classifying subscapularis atrophy.

## Methods

In this retrospective case-controlled study, 49patients who underwent MRI imaging of the shoulder between 2007 and 2014 in this institution without rotator cuff pathology, were selected at random as a control group. Exclusion criteria were glenohumeral disorders (labral/SLAP lesions or instability etc.), osteoarthritis, full or partial thickness rotator cuff tear, atrophy or fatty infiltration of any rotator cuff muscle, humeral head migration, neurologic disorders involving the shoulder girdle or any previous shoulder surgery, as well as prolonged duration of pain or immobilization. This study was approved by the institutional ethics committee and informed consent was obtained from all patients.

The study group comprised 11 patients presenting with isolated SSC pathology and muscular atrophy, between December 2002 and November 2007. Standardized MRI examinations were performed with the arm in neutral position on a 3.0-Tesla system (Verio; Siemens Medical Solutions) with use of a dedicated phased-array shoulder coil. T1 and T2-weighted sequences in axial, coronal and oblique-sagittal planes were recorded. Subscapularis atrophy was assessed in the oblique sagittal plane [2, 16, 25, 33, 35, 37].

SSC muscle atrophy was analyzed by two blinded examiners, one radiologist specializing in musculoskeletal imaging and one experienced orthopedic shoulder specialist. All values were calculated independantly and the mean value taken for definite analysis. In addition, inter-observer reliability was assessed.

### Subscapularis diameters and ratio (cranial-transverse/caudal-transverse)

Maximum vertical and transverse (cranial-transverse and caudal-transverse) diameters were calculated in millimeters in the same plane, as described by Scheibel et al. [30]. Measurements are performed on parasagittal images, using the most lateral image on which the spine of the scapula is in contact with the coracoid process (Y-shaped position). The vertical diameter is defined as the distance between the highest and the lowest point of the subscapularis muscle. The measuring line of the cranial-transverse diameter is placed perpendicular to the vertical diameter, ending at the top of the concavity of the subscapularis groove. The maximal caudal-transverse diameter was also measured perpendicular to the vertical diameter and ends at the most inferior point of the scapula.

### Cross sectional area (CSA) and its ratio (upper half/total muscle)

**Fig. 1:**
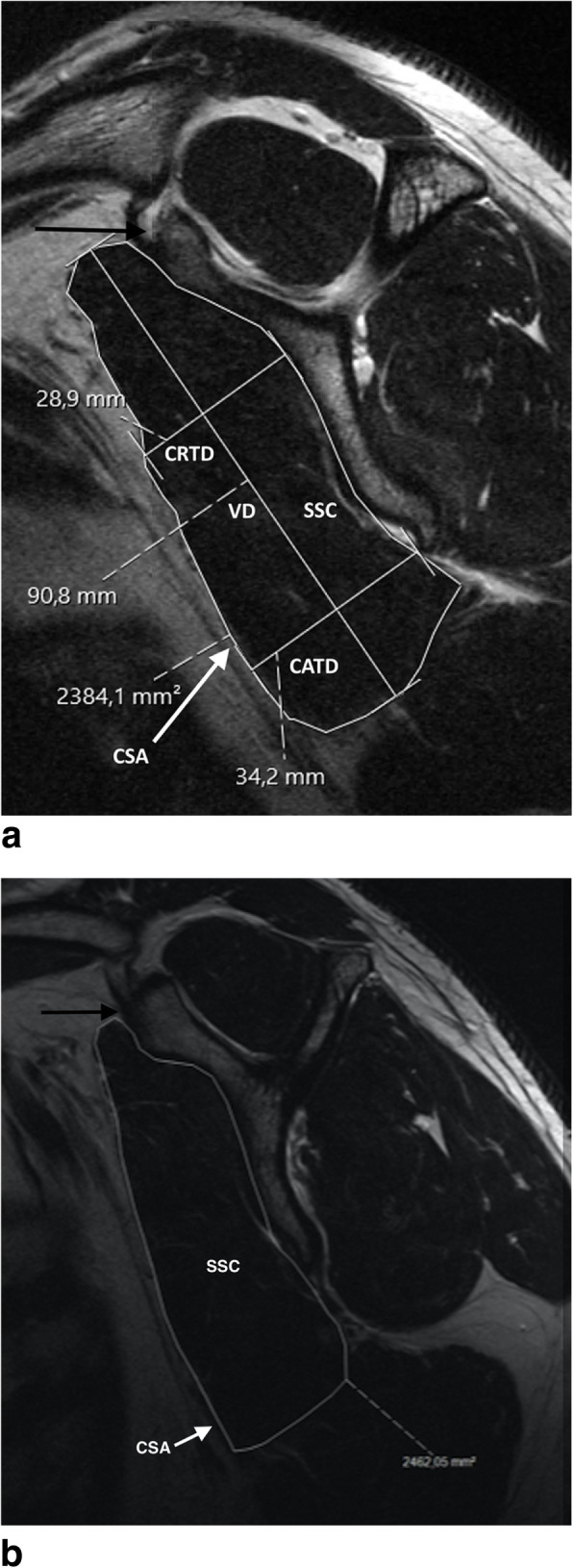
**a** Healthy subscapularis muscle - measuring of the vertical diameter (VD), cranial transverse diameter (CRTD), caudal transverse diameter (CATD) and cross sectional area (CSA); SSC: subscapularis muscle; black arrow: ventral tip of the scapular “Y”. **b** “Y-position” of the MRI sagittal oblique plane: Healthy subscapularis muscle - measuring of the cross sectional area (CSA); SSC: subscapularis muscle; arrow/CSA: cross sectional area; black arrow: ventral tip of the scapular “Y”

The cross-sectional area of the SSC was measured in the “Y-position” (using the most lateral image where the scapular spine is in contact with the body of the scapula) of the MRI sagittal oblique plane. By this means, a standardized view can be guaranteed for reproducible calculation. The CSA was measured using manual tracing of the SSC muscle outline on the PACS workstation and specified in square millimeters (mm^2^) based on the technique of Juul- Kristensen et al. [17] and modified by Bartl et al. [3] (Fig. 1).

**Fig. 2:**
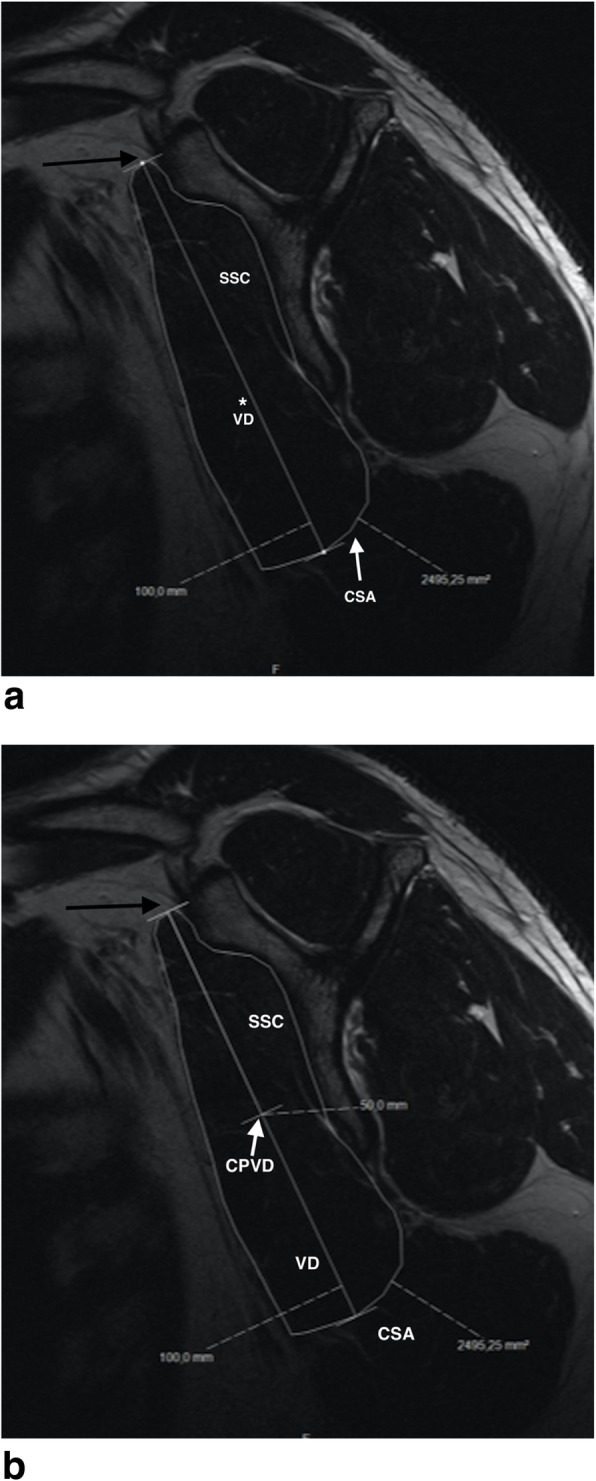
**a** Healthy subscapularis muscle - measuring of the vertical diameter (VD): SSC: subscapularis muscle; asterisk/VD: vertical diameter; arrow/CSA: cross sectional area; black arrow: ventral tip of the scapular “Y”. **b** Healthy subscapularis muscle - determining the center of the vertical diameter; SSC: subscapularis muscle; VD: vertical diameter; arrow/CPVD: center point of the vertical diameter; CSA: cross sectional area; black arrow: ventral tip of the scapular “Y”

To determine the upper 50% of the subscapularis area in the same plane, the center of the vertical diameter was calculated (Fig. 2a and b).

**Fig. 3:**
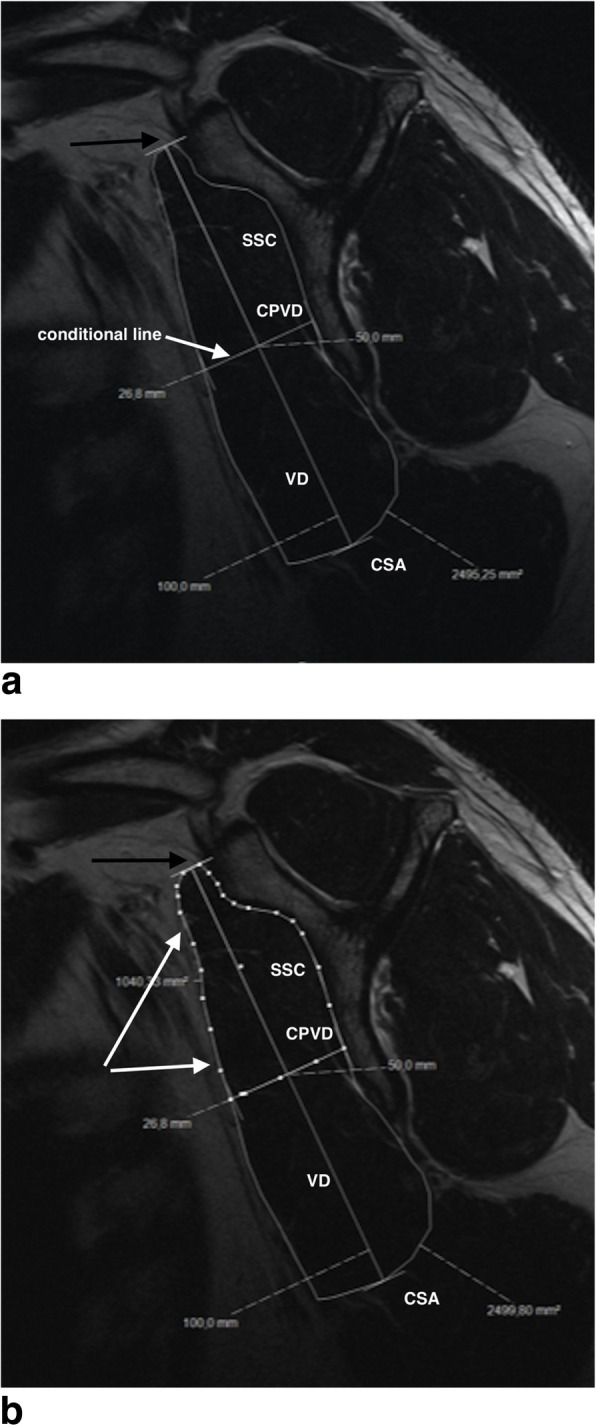
**a** Healthy subscapularis muscle - conditional line at the center point of the vertical diameter; SSC: subscapularis muscle; VD: vertical diameter; CPVD: center point of the vertical diameter; CSA: cross sectional area; white arrow: conditional line perpendicular to the vertical diameter to define the upper half of the subscapularis cross sectional area at the midpoint of the vertical diameter; black arrow: ventral tip of the scapular “Y”. **b** Healthy subscapularis muscle - defining the upper half of the subscapularis CSA; SSC: subscapularis muscle; VD: vertical diameter; CPVD: center point of the vertical diameter; CSA: cross sectional area; white arrows: definition of the upper half of the subscapularis cross sectional area at the center point of the vertical diameter

At this point a conditional line was created perpendicular to the vertical diameter to define the upper half of the subscapularis cross sectional area at the midpoint of the vertical diameter (Fig. 3a and b). Using these data, the ratio of the CSA of the upper half relative to the total CSA of the SSC was calculated.

### Cross sectional area (CSA) with defined bony vertical landmark and its ratio (upper half/total muscle)

In all 49 patients of the reference group, the subscapularis muscle, determined at the “Y-shaped position”, was cranially bounded at its upper margin by the bony ventral tip of the scapular “Y” and never extended directly to the coracoid (Fig. 2).

Given this observation, the ventral tip of the scapular “Y” represents a bony margin of the subscapularis muscle and may be used as osseous landmark of the vertical diameter to evaluate the cranial extent of the SSC, particularly in patients with marked atrophy. In patients with physiological subscapularis muscle bulk, without atrophic changes, the cranial extent of the SSC seems to be equivalent to the measuring method described by Scheibel et al. [30] assessing the vertical, the cranial-transverse and the caudal-transverse diameters of the SSC.

**Fig. 4:**
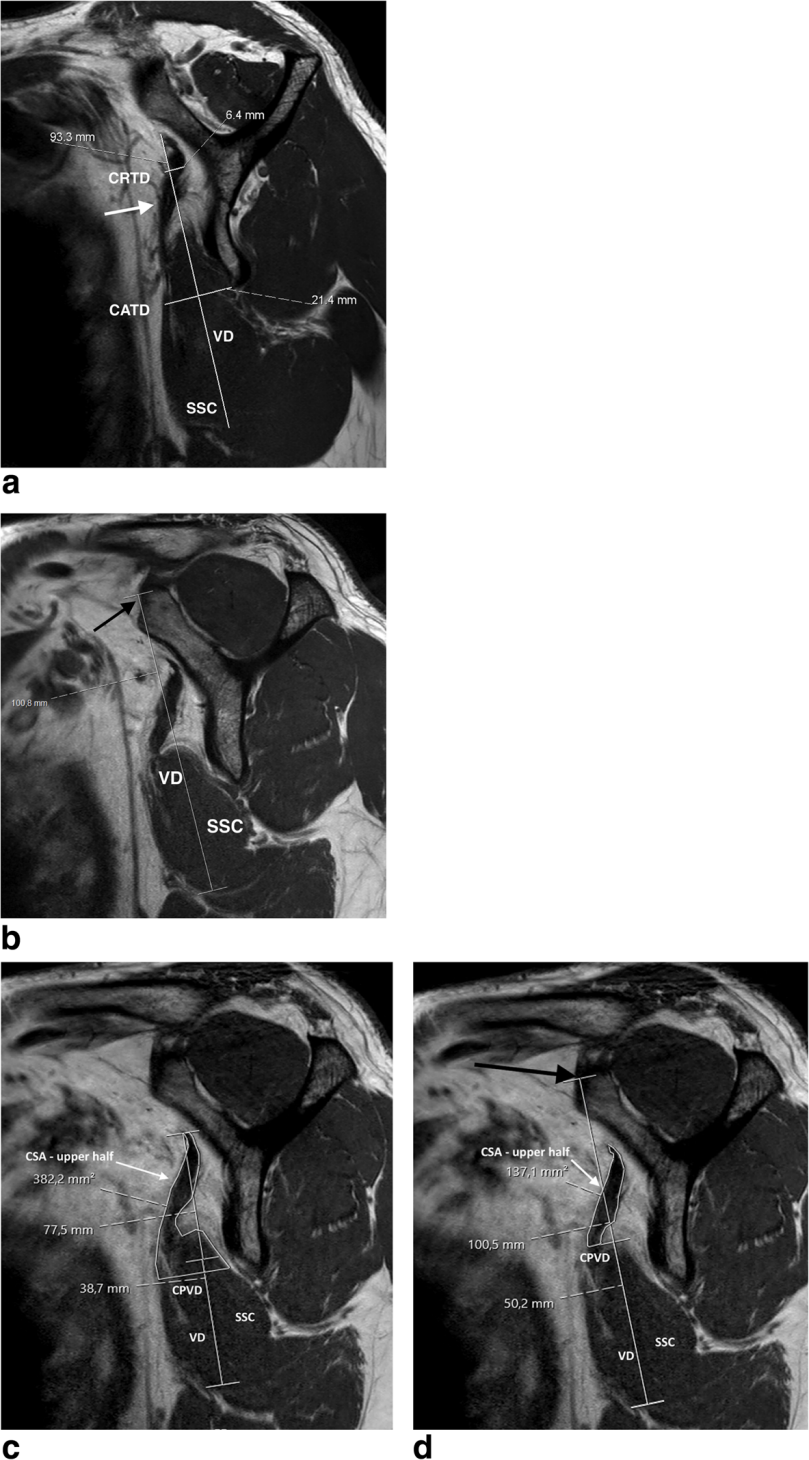
**a** and **b** Atrophic subscapularis muscle - measuring of the vertical diameter without (**a**) and with (**b**) bony landmark; SSC: subscapularis muscle; white arrow: cranial atrophy of the SSC; VD: vertical diameter; CRTD: cranial transverse diameter; CATD: caudal transverse diameter; black arrow: ventral tip of the scapular “Y” as bony landmark to define the bony referenced VD. c and d: Atrophic subscapularis muscle - measuring of the cross sectional area of the upper half of the SSC muscle without (**c**) and with (**d**) bony landmark; SSC: subscapularis muscle with cranial atrophy; VD: vertical diameter; black arrow: ventral tip of the scapular “Y” as bony landmark to define the bony referenced VD; white arrow: CSA (cross sectional area) of the upper half of the muscle; CPVD: center point of the vertical diameter

In contrast, particularly in patients with marked cranial SSC atrophy, this difference (real cranial vertical diameter tip vs. defined bony landmark – (Fig. 4 and)) may represent a significant bias in measuring CSA ratio and therefore in evaluating SSC atrophy given that the ratio of the upper half of the SSC in relation to the total muscle alters dependent on the vertical diameter measured.

The CSA (with and without defined bony cranial landmark) and its ratios as well as the muscle diameters of the 49 control scans, as well as the 11 patients with subscapularis atrophy, were compared for statistical difference.

## Statistics

With a sample size of 11 patients with atrophy and 49 controls, the study was adequately powered (> 80%) to detect an effect size (Cohen’s d = mean difference between groups / standard deviation within groups) of one. Power calculation was performed with nQuery Advisor 7.0.

Statistical analysis was performed using SPSS software version 24.0 (IBM corp, Armonk, New York, USA) and R version 3.3.1 (R Foundation for Statistical Computing, Vienna, Austria). Correlations of assessments by the raters were calculated using the Pearson correlation coefficient. Correlation coefficients were compared using Dunn and Clark’s test provided in the R package *cocor* [6]. To evaluate differences between the control and the atrophy group diameters, cross sectional areas and the CSA ratio of the subscapularis muscle, the ROC curve was determined. Measurement of the CSA ratio bony referenced vs. not bony referenced was compared by the Bland-Altman procedure. All statistical tests were performed two-tailed and the level of significance was set at *p* < 0.05.

## Results

The control group comprised 33 male and 16 female participants (*n* = 49) with a mean age of 49.7 (range, 33,6 – 69,2).

SSC diameters were 9.3 cm ± 1.5 (vertical), 2.7 cm ± 0.6 (cranial-transverse) and 3.1 cm ± 0.8 (caudal-transverse), respectively. The diameter ratio (cranial-transverse (caudal-transverse) was 0.886 ± 0.159.

The mean cross-sectional area of the SSC calculated conventionally was 2318.0 mm^2^ (± 743.0) for the complete subscapularis muscle and 1021.3 mm^2^ (± 292.7) for the upper half. The subsequent mean CSA ratio was 0.448 (± 0.052).

Inter-rater reliability demonstrated a correlation of 0.985.

By calculating the cross sectional area (CSA) defined by cranial bony landmarks, the mean total area and area of the upper half of the SSC was 2367.0 mm^2^ (± 741.4) and 1048.2 mm^2^ (± 313.3), respectively. The resulting mean ratio (upper half/total muscle) was 0.446 (± 0.046). Inter-rater reliability demonstrated a correlation of 0.999. The mean vertical diameter was 9.0 cm (± 1.5).

Comparing these two methods of calculating CSA ratio, it was seen that a significantly more accurate and representative measurement may be achieved by use of the bony-referenced method (*p* = 0.001).

The atrophy group consisted of eight male (72.7%) and three female (27.2%) patients (*n* = 11) with a mean age of 56.7 (range, 44,0 - 71,7).

Calculated conventionally, diameters were 7.6 cm ± 0.8 (vertical), 1.0 cm ± 0.7 (cranial-transverse) and 2.8 cm ± 0.6 (caudal-transverse), respectively. The diameter ratio (cranial-transverse/caudal-transverse) was 0.355 ± 0.282.

The mean cross sectional area was 1572.4 mm^2^ (± 411.8) for the complete and 510.0 mm^2^ (± 173.1) for the upper half of the subscapularis muscle. The mean CSA ratio of 0.322 (± 0.056) was significantly reduced as compared to the control group (0.448 ± 0.052, *p* = 0.001). The area under the ROC curve (AUC) was 0.99.

Inter-rater reliability showed a correlation coefficient of 0.98.

Using the bony-referenced method, the osseous-bounded vertical diameter was 8.4 cm ± 0.8. The CSA of the 11 patients with atrophy showed mean values of 1554.7 mm^2^ (± 419.9) for the total muscle and 422.9 mm^2^ (± 173.6) for the upper half, both significantly reduced as compared to the control group (total CSA 2367.0 mm^2^ (± 741.4), upper half CSA 1048.2 mm^2^ (± 313.3), *p* = 0.001).

This also resulted in a significant reduced mean CSA ratio (0.269 ± 0.065) in the atrophy group as compared to the control group (p = 0.001) and perfect discrimination was shown in the ROC curve with an AUC value of 1. Inter-rater reliability showed a correlation of 0.99.

**Fig. 5:**
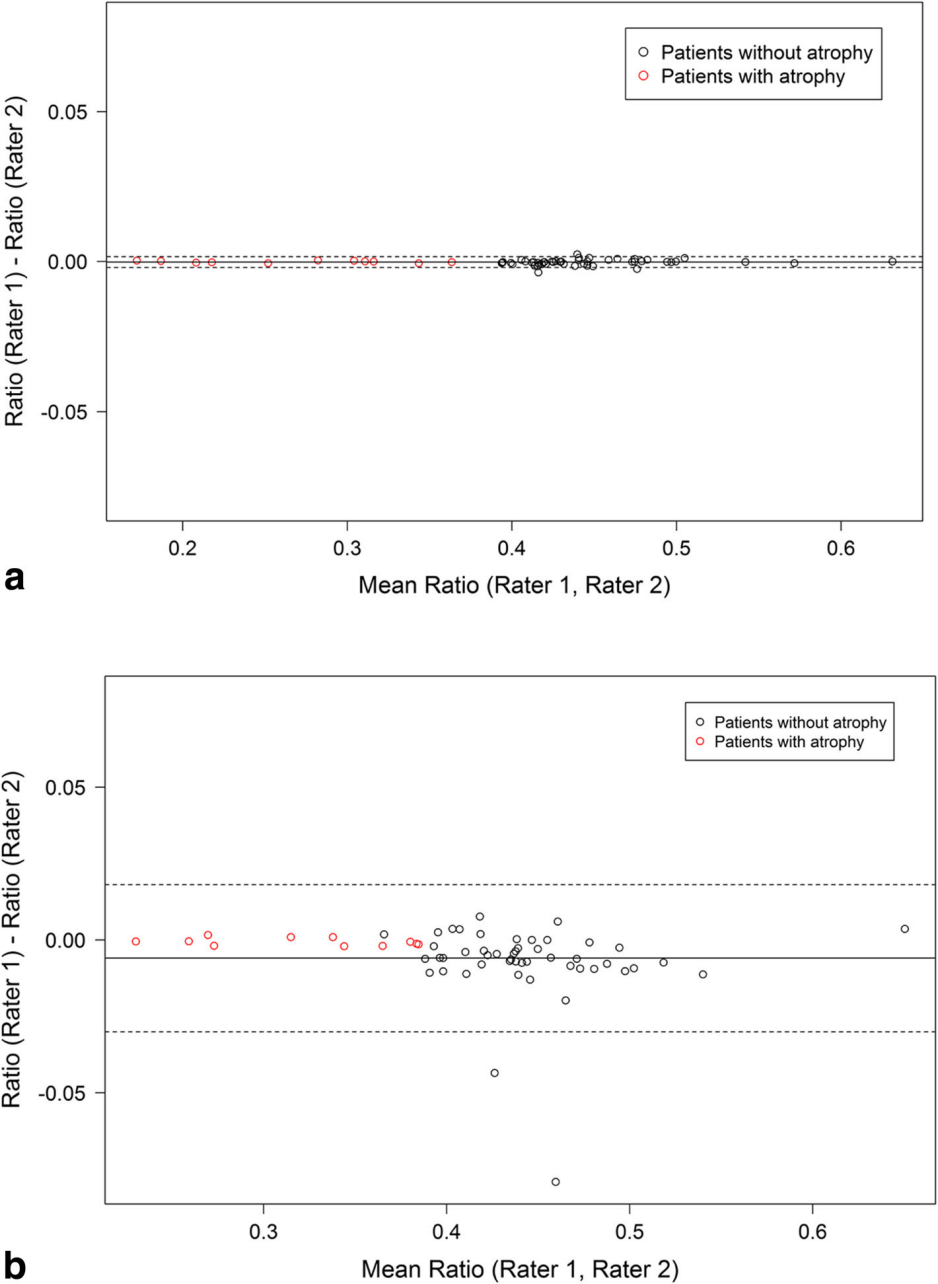
**a** Inter-rater reproducibility regarding the CSA ratio of the atrophy and the control group – with bony landmark. **b** Inter-rater reproducibility regarding the CSA ratio of the atrophy and the control group – without bony landmark

When comparing CSA ratio values for the conventional versus the bony referenced method in both groups, significantly more accurate and reflective measurements may be achieved by use of the bony referenced method (p = 0.001) (Fig. 5a and b).

Assessing all measured and calculated parameters (CSA ratio, CSA of the upper half and the total muscle as well as the three diameters) for capacity to quantify subscapularis atrophy, the CSA ratio represents the most reliable tool with an area under the curve (AUC) of 1.00 when using the bony-referenced method, and 0.984 using the conventional method (*p* < 0.001).

Based on these data, we propose that subscapularis atrophy be graded into a four-stage classification:

- Stage 0: no atrophy; CSA ratio > 0.4
- Stage I: mild atrophy; CSA ratio < 0.4–0.35
- Stage II: moderate atrophy; CSA ratio < 0.35–0.3
- Stage III: severe atrophy; CSA ratio < 0.3

According to this classification, subscapularis atrophy with bony landmark could be graduated as mild (Stage I) in 1 out of 11 (9.1%) patients. Four (36.4%) patients had moderate atrophy (Stage II) whereas severe atrophy (Stage III) could be detected in six (54.5%) patients (see Table 1).

**Table 1:**
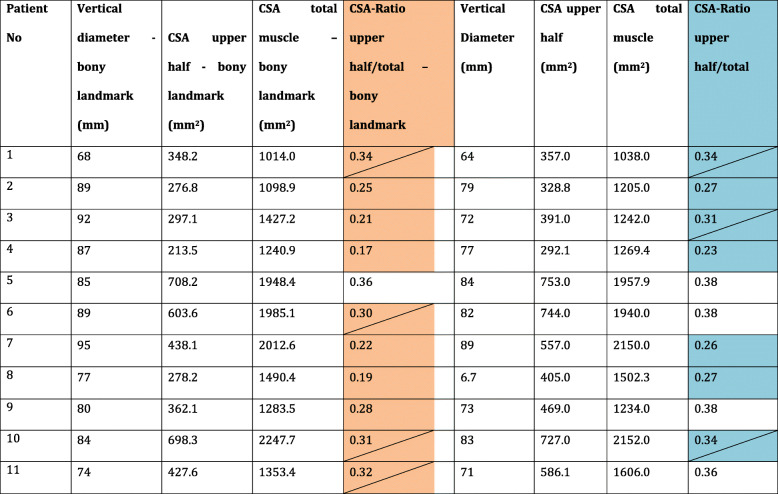
Patients Demographics – Atrophy group

Orange: Patients with severe atrophy (bony referenced CSA ratio < 0.30): *n* = 6; Orange with diagonal line: moderate atrophy (bony CSA ratio < 0.35–0.30): *n* = 4

Blue: Patients with severe atrophy (conventional CSA ratio < 0.30): *n* = 4; Blue with diagonal line: moderate atrophy (conventional CSA ratio < 0.35–0.30): *n* = 3

## Discussion

The present study demonstrates that subscapularis atrophy may be quantified by measurement of the CSA ratio using a “Y”-view MRI slice, and classified into a four-stage grading system proposed here.

Analyzing atrophy patterns of the subscapularis muscle after reconstruction of isolated subscapularis tendon tears, it has been shown that SSC atrophy always occurs from the top down [15, 28]. The cranial aspect of the SSC seems to be affected the most whereas isolated atrophy within the caudal area of the SSC was not seen in the present cohort nor has been described in the literature previously.

Evaluating the normal MRI images of the 49 control patients, it was seen that the SSC never extends beyond the coracoid process in the oblique sagittal plane. This ventral tip of the scapular “Y” may therefore be considered a bony landmark of the maximal cranial extension of the subscapularis muscle.

While classifications of muscular fatty infiltration are well described by Goutallier et al. [12] by means of computerised tomography (CT) and by Fuchs et al. [9] using MRI, grading systems of muscular atrophy remain rare. Thomazeau et al. [35] and Zanetti et al. [38] published classification systems for supraspinatus atrophy and Warner et al. [37] described a method of classifying combined antero-superior rotator cuff atrophy. In their study Warner et al. [37] introduced a measurement of muscle atrophy based on oblique sagittal plane image medial to coracoid process. The stage is specified by the amount of muscle above or below a line drawn from the edge of the coracoid to the inferior tip of the scapular spine. In addition, the Zanetti tangent line connects the superior aspect of the coracoid and the scapular spine. A convex overhang of the muscle above the line indicates no atrophy and a decrease of the muscle area towards the line or a concavity below the line represents mild, moderate or severe atrophy, respectively. No quantification of atrophy was presented.

Thomazeau et al. [35] used the supraspinatus fossa as osseous landmark to define SSP atrophy. Contrary to the SSP there is no three-side bony limitation of the SSC to guarantee three-side reproducible measurements. Defining osseous landmarks in calculating subscapularis atrophy is challenging and may explain the previous absence of a standardized SSC atrophy grading system.

Schröder et al. [31] and Scheibel et al. [30] semi-quantitatively evaluated atrophy of the subscapularis by measuring the vertical, the cranial-transverse and the caudal-transverse diameters. In addition, the signal to noise ratio of the SSC and the ISP were examined, although without proposing any grading of muscular atrophy. In patients with massive atrophy, usually appearing in the upper part of the muscle, this method may become insufficiently accurate as the cranial-transverse diameter is often difficult to determine due to loss of volume. This potential issue is corroborated by the observations of the present study. Therefore, a reliable determination of atrophy exclusively based on the above mentioned three diameters (vertical, cranial-transverse and caudal-transverse) may be inadequate. Likewise, assessing subscapularis atrophy by comparing the signal to noise ratio of the subscapularis and the infraspinatus muscle may be skewed by the requirement for a non-atrophic infraspinatus muscle.

Atrophy of the subscapularis muscle is commonly seen in the upper part, theorised to be a result of the tendency of the subscapularis tendon to ruptures from the top down, as the inferior part of its humeral insertion is of muscular origin and not tendinous. For this reason, it is useful to evaluate the upper part as compared to the total muscle.

Although isolated caudal lesions of the subscapularis tendon [1, 14, 18] have been, rarely, described in literature, there are no reports of isolated atrophy of the lower part of the SSC. In the present study, caudal atrophy of the SSC was not seen in any of the 11 patients with subscapularis atrophy. It may therefore be assumed that the distal border of the SSC, even in case of higher grades of atrophy, is unlikely to change position significantly.

Given these findings, determining the cross-sectional area seems to be a more reliable method to assess subscapularis atrophy, particularly when using a defined plane in the oblique sagittal “Y-View” (using the most lateral image where the scapular spine is in contact with the body of the scapula). As each patient has a unique subscapularis cross sectional area (atrophic or not), calculating the CSA ratio of the upper part in relation to the total muscle seems to represent a reliable and reproducible tool to assess SSC atrophy.

Using conventional methods, defining the maximal vertical spread from the base to the preserved top of the subscapularis, biased CSA ratios (upper half of the SSC in relation to the total muscle) may result in mild atrophic muscles as compared to severely atrophic cases (see Table 1).

Analyzing the data and MRI images of the control group it was seen that healthy, non-atrophic SSC muscles never extend the coracoid process. Thus, it is proposed to use the ventral tip of the scapular “Y” as a defined osseous landmark for the upper margin of the subscapularis muscle in calculation of the CSA ratio to ensure easily reproducible measurements. This was supported by the good correlation (correlation coefficient: 0.99 - bony referenced) between the two independent examiners determining the CSA ratio in this study.

The major difference between the CSA ratio and the CSA ratio with bony landmark is that the area of the upper half in relation to the total muscle decreases by taking the original vertical diameter for reference (see Fig. 4c and d). Thus, the centerline between the upper and the total muscle is translated cranially and the area of the upper muscle part decreases. In case of (severe) muscle retraction subscapularis measurement with bony landmark may also constitute a more accurate way of evaluation – provided that measurement is performed at a defined sagittal oblique plane of the MRI (“Y-position”). A severe retracted tendon is supposed to lead to a decrease of the CSA ratio, too.

This observation was confirmed by analysis of the measurements of the atrophy group (see Table 1). Compared to the conventional, non-bony-referenced measurement, (mean value 510.0 ± 173.1) the area of the upper part of the SSC was significantly decreased (*p* < 0.001) in all patients by using the osseous landmark measurement technique (mean value 422.9 ± 173.6). Patients of the atrophy group also had a significantly (p < 0.001) reduced bony landmark CSA ratio with mean values of 0.269 ± 0.065 as compared to the conventionally measured CSA ratio with mean values of 0.322 ± .056. However, it has to be stated that both groups are of limited size. Due to the reason that isolated subscapularis tendon tears only represent a very small part of all rotator cuff tears there is still a lack of studies presenting data regarding isolated subscapularis tears in lager numbers of patients [3, 13, 20, 22] – especially regarding subscapularis atrophy.

Comparing all parameters (three diameters, CSA and CSA ratio) regarding capacity to quantify subscapularis atrophy, the bony referenced CSA ratio represents the most reliable tool with an area under the curve (AUC) of 1.00 (*p* = .0001).

Based on the bony referenced CSA ratio, a quantitative classification of subscapularis atrophy is proposed as follows:

Stage 0 with a ratio of > 0.4 represents no atrophy and is also seen in healthy patients. Mild atrophy with a CSA ratio < 0.4–0.35 is classified as Stage 1. Stage 2 indicates moderate atrophy with ratio values < 0.35–0.3. Values < 0.3 represent severe atrophy of the subscapularis.

A reliable and reproducible pre-operative analysis of isolated subscapularis atrophy in cases of SSC rupture may assist decision-making and inform choice of treatment and feasibility of subscapularis repair. Postoperatively, it may be used to monitor healing and evaluate clinical outcome.

To confirm this, further prospective combined clinical-radiological studies will be necessary to validate whether quantitative pre-operative assessment of subscapularis atrophy is a useful prognostic indicator of postoperative outcome.

## Limitations

There are some limitations to the present study which must be considered. Firstly, the number of patients within the control group, acting as anatomical “normal” examples, and within the atrophy group is limited.

Secondly, the differences in the mean age between the study and the control group may act as a bias. Additionally, functional parameters such as clinical tests, strength, subjective satisfaction or pain evaluation are missing in the atrophy group to draw further clinically related conclusion, although the 11 patients in this cohort nonetheless acted well to illustrate the significant differences in the CSA ratio as compared to the control group. Prospective clinical studies with larger numbers of patients and separate cohorts, including clinical parameters and equal gender distribution, are needed to confirm our findings. In addition, the atrophy group consists of patients with isolated subscapularis atrophy. It would be interesting to see if there are any changes on muscular atrophy over the course of time.

Furthermore intra-rater reliability is missing.

For this reason a more comprehensive study including clinical parameters is being undertaken to provide clinical correlation and to allow recommendations to be made regarding treatment options, on the basis of preoperative MRI evaluation of subscapularis atrophy.

## Conclusion

Analyzing atrophy patterns in cases of isolated subscapularis tears it was demonstrated that the bony referenced cross sectional area ratio (area of the upper half in proportion to the total muscle) may represent a reliable and reproducible method of quantifying and subsequently classifying isolated subscapularis atrophy, although data regarding subscapularis atrophy are limited as isolated subscapularis tears represent a very rare entity.

## Data Availability

All relevant data supporting the conclusions are included within the article and tables. The raw data are part of a greater institutional investigation project. The datasets used and/or analyzed during the current study are available from the corresponding author on reasonable request.

## Abbreviations

CATD: Caudal transverse diameter
CPVD: Center point of the vertical diameter
CRTD: Cranial transverse diameter
CSA: Cross sectional area
SSC: Subscapularis muscle
VD: Vertical diameter
